# From across the Seas

**Published:** 1917-08-25

**Authors:** 


### FROM ACROSS THE SEAS.

### Royal Prince Alfred Hospital.

The March number of the *Royal Prince Alfred Hospital Gazette*, Sydney, is used chiefly as a medium for presenting the annual report of the hospital. In 1916, with a capacity of 410 beds, the average daily occupation was 392, and the number of attendances in the out-patient and casualty departments was 118,945. The percentage number of operations to admissions has risen from 13.75 in 1883 to 78.44 in the year under review. The hospital has experienced great difficulty in obtaining an adequate supply of drugs and other requirements for the treatment of patients. The number of students who attended the practice of the hospital was very high, though the total was not quite so great as it has been in some years immediately preceding the war. Nursing difficulties have naturally arisen, but the report testifies that the work of the staff was carried out with smoothness and efficiency. Both the revenue and expenditure showed a material expansion; in view of the greater number of beds under occupation and of out-patients the Government grant in respect of the treatment of venereal cases was increased from £500 in 1915 to £1,700 in 1916, and in the same period the contributions by in-patients rose from £5,620 to £6,511. The system of joint purchase of certain supplies by the various Metropolitan hospitals has proved so satisfactory that the arrangement has been renewed for another year. The general Government subsidy of the hospital now stands at £14,000 a year, and the total expenditure in 1916 was £53,023.

### The Tasmanian Hospital Difficulty.

From the *Medical Journal of Australia*, Sydney, we learn the sequel to the difficulty that has arisen between the consultants and the State. As will be remembered, the Council of the Tasmanian branch of the British Medical Association informed the Premier that unless an assurance were given that persons in affluence would be excluded from the State-aided hospitals, the honorary medical officers would be forced to tender their resignations. The letter conveying this message bore the date of February 15, 1917, and no reply was received until March 17, when the Premier wrote to the effect that he had discussed the matter with his colleagues in full Cabinet meeting. No decision had been arrived at, and he informed the Council that the matter needed careful consideration. "Pending such consideration, it is not intended to make any alterations in existing conditions." The Council decided to act at once, and called upon all honorary medical officers on the staffs of the State-aided hospitals in Tasmania to forward their resignations to the honorary secretary of the branch by March 26, 1917. Every practitioner holding these positions complied with the request, and the honorary secretary was able to forward to the secretaries of the hospitals the resignations of all the honorary medical officers.

### An Outspoken Colonial Secretary.

The Colonial Secretary, Mr. H. P. Colebatch, has frankly told the citizens of Fremantle, Western Australia, that they are not doing enough to support their own hospital. The occasion was one of those frequent occurrences when an Australian hospital board asks for an increased Government grant; in this case it was a deputation from the Fremantle Public Hospital who made an application. The Minister said he desired to express at the outset the recognition by the Government of the excellent work the board had done in administering the hospital, but in respect of the financial side of the situation he stated that in Perth and Fremantle the idea had grown up that at these public hospitals people were entitled to receive free medical treatment whether they can afford to pay or not. Whilst the hospitals of both cities were asking for increased subsidies, he was compelled every week to refuse requests from other communities who were cheerfully paying up to three-quarters of their hospital maintenance. If the people of Fremantle wished to have their hospital, they must do a reasonable thing to maintain it. He advised the board that much greater pressure must be brought to make patients, in a position to do so, suitably to contribute towards the cost of their maintenance and treatment.

### The Family Doctor's Influence in Charity Work.

An interesting article in the *Medical Record*, New York, gives many instances of charitable bequests and gifts influenced by the family physician, and argues how well-placed he is to secure the direction of benefactions into good channels. One example cited is that of a plain, retiring physician who obtained the confidence of a wealthy visitor to the neighbourhood. The rich man had a family growing up in idleness without an incentive to work, and getting ready to spend the father's laboriously acquired wealth to their ultimate destruction. The plain physician pointed out the probable consequences and persuaded the wealthy man to put responsibility upon his family and give away his property very largely to real charities. The result was that he founded a great hospital and left many endowments and built up a spirit of benevolence in his family, who sought to give money away rather than spend it on themselves. Here the physician was an active force in changing the destinies of this man and his family and giving them an enviable place in the community; otherwise they would have gone the way of others. A well-known doctor once remarked, "I believe I have done more to benefit my patients by suggestions and general counsel to individuals than by any drugs I have ever given." He was right, says the writer of the article. The spirit of accomplishing something can be fostered, encouraged, and directed by physicians into this new, unknown world.

